# The Meeting at Minneapolis

**Published:** 1885-09

**Authors:** 


### THE MEETING AT MINNEAPOLIS.

The late meeting of the American Dental Association was, in many respects, the most successful that has been held in years. First, the attendance was larger, over one hundred new members having been secured. Second, the papers were generally of a better character than usual, and the discussions were more intelligent and erudite. Finally, the social aspect was exceedingly pleasant, for the hospitality of the western dentists is as broad as their prairies, their love for their professional brethren as deep and as pure as the waters of their lakes. When one looks upon the teeming richness of their soil, he cannot but believe that they live directly upon the breast of mother earth, nearest the fount of nourishment, and, therefore, it should be no matter of surprise if they were able to exhibit the strongest, bravest men, and the fairest, truest women, as well as the biggest pumpkins and greatest fields of corn.

It would not be impossible successfully to criticise some things, and perhaps more benefit will be derived from a pointing out of faults to be avoided, than from ill-deserved and indiscriminate praise. There was altogether too much of school-boy talk, but probably from an Association formed of such heterogeneous elements it would be impossible to eliminate all crudities. There are too many vain members, who speak simply that their names may be published in the reports as having taken part in the discussions, and who thus worse than waste valuable time. It is not an exhibition of which the representative American Dental Society should be proud, when members who should have made greater advances gravely proceed to ruminate upon the most elementary principles, and thus lead a discussion, that might otherwise take a higher range, into a consideration of mere puerilities and commonplace truisms. The members of a National Society are supposed to have advanced beyond mere scientific rudiments.

Those who have been in regular attendance upon the meetings of the A. D. A. cannot have failed to notice a constant growth. It is not a really scientific body as yet, but it bids fair to become such, when it learns sternly to frown down mere glittering generalities and empty rhetoric, and to view every subject from a real, and not a pseudo-imaginary scientific, stand-point.

The meeting of this year has taught that it is well occasionally to hold the sessions at extreme points of the country. It would not be safe to do this two years in succession, because if the meetings be for a number of consecutive years held in the west, it is not only a hardship to the eastern men, but it causes them soon to lose their interest in the Association. Had the question been submitted this year, it would have been easy, in a meeting composed so largely of western men, to have elected another western point for the next meeting, and thus to have given rise to a charge of sectionalism, but it was probably wisely left to the discretion of the Executive Committee, with power to call the next meeting at the point which shall seem to them to promise the best general attendance.

It was a matter of congratulation that the constitution tinkers and the by-law debaters were not out in full force, and therefore there was considerable time left for the more legitimate objects of the meeting. In fact, there was less of professional politics than for some years past. Those who consider it a smart thing to manipulate a scientific body, who attend for the mere purpose of pulling the wires, were either engaged in other business, or were absent altogether. There was something too much of it as it was, but it was never offensively exhibited, nor was it allowed to interfere with legitimate work. It is to be hoped that we have seen the last of the wire-pullers.

The work of the Executive Committee was much more quietly done than is usual, but it was certainly as effectually performed. At some of our meetings the business committees have used up a good deal of time and created very much of confusion in their futile attempts to expedite matters. At Minneapolis the committees seemed to catch the full spirit of their mission, which is to see that affairs are conducted without friction, and not to be continually interrupting business by officious interference.

The President, Dr. J. N. Crouse, firmly insisted upon holding the Society strictly down to the questions under consideration, although he used up his gavel and nearly battered down his desk in his persistent efforts to keep order in an unruly assemblage. As a very fitting testimonial to his unremitting efforts, the Society presented him with the remnants of the gavel, first giving directions that it should be properly repaired, ornamented and inscribed.

